# The completeness of cancer registration in England: an assessment from the Oxford-FPA contraceptive study.

**DOI:** 10.1038/bjc.1988.252

**Published:** 1988-10

**Authors:** L. Villard-Mackintosh, M. P. Coleman, M. P. Vessey

**Affiliations:** Department of Community Medicine & General Practice, University of Oxford, UK.

## Abstract

The completeness of cancer registration in England for the period 1968-85 has been assessed in a cohort of 17,000 women who reported malignancies directly to the investigators. Of 325 cancers reported, 281 (86.5%) had been registered by mid-1987. Under-registration varied considerably between regional cancer registries. Eight (18%) of the 44 unregistered cancers were treated in private hospitals. Under-registration also varied considerably with cancer site: only 8% of 150 breast cancers were not registered, and at sites accounting for 79% of all tumours, under-registration was less than 15%; however, 40% of melanomas (20 cases) and 50% of lung cancers (6 cases) were not registered. Of 281 registered tumours, only 219 (78%) were notified to the investigators from the NHSCR at Southport, with a median lag-time of 2.5 years since diagnosis. There has been a tendency for notification of registered cancers to the investigator to become more prompt but less complete.


Br. J. Cancer (1988), 58, 507–511 © The Macmillan Press Ltd., 1988

### The completeness of cancer registration in England: an assessment from the Oxford-FPA contraceptive study

L. Villard-Mackintosh[1], M.P. Coleman[2] & M.P. Vessey[1]

[1]*Department of Community Medicine & General Practice, University of Oxford, Gibson Building, Radcliffe Infirmary, GB Oxford OX2 6HE, UK and* [2]*Unit of Descriptive Epidemiology, International Agency for Research on Cancer, 150, cours Albert Thomas, F 69372 Lyon Cedex 08.*

**Summary** The completeness of cancer registration in England for the period 1968–85 has been assessed in a cohort of 17,000 women who reported malignancies directly to the investigators. Of 325 cancers reported, 281 (86.5%) had been registered by mid-1987. Under-registration varied considerably between regional cancer registries. Eight (18%) of the 44 unregistered cancers were treated in private hospitals. Under-registration also varied considerably with cancer site: only 8% of 150 breast cancers were not registered, and at sites accounting for 79% of all tumours, under-registration was less than 15%; however, 40% of melanomas (20 cases) and 50% of lung cancers (6 cases) were not registered.

Of 281 registered tumours, only 219 (78%) were notified to the investigators from the NHSCR at Southport, with a median lag-time of 2.5 years since diagnosis. There has been a tendency for notification of registered cancers to the investigator to become more prompt but less complete.

Malignant neoplasms arising in a cohort under investigation in England may be ascertained through contact with members of the cohort, with the employer or organization from which the cohort is derived, with one or more of the 11 regional cancer registries covering the population, or by using a combination of these methods. However, the National Health Service Central Register (NHSCR) for England and Wales, based at Southport, can also notify *bona fide* investigators of cancer registrations and deaths among members of a defined cohort. After approval of the study protocol by the British Medical Association's Ethical Committee and by the Office of Population Censuses and Surveys (OPCS), the NHSCR record of each individual in the cohort can be marked with a code indicating membership of the study cohort, ('flagged'). In theory, the NHSCR receives copies of all death certificates and, since 1971, all cancer registrations, to enable cancer survival statistics to be generated (Balarajan & Scott, 1983); when a death certificate or a cancer registration relates to a flagged individual, the investigator is notified (notifications are made at quarterly intervals). About 96% of the 200,000 cancer registrations in England and Wales each year are successfully traced to an individual's record at NHSCR (Swerdlow, 1986), and provided all the members of a cohort can be similarly traced for flagging of their records, virtually complete ascertainment of cancer registrations and deaths in the cohort should be assured.

About 200 studies have used this system since 1971. There has recently been some interest in assessing its efficiency in practice, since the accuracy of investigations which depend solely on the NHSCR for information on cancer incidence and death may be seriously affected by incompleteness of regional cancer registration, and by delays or errors in the various stages between registration and notification. A recent study of 50 confirmed breast cancers in a flagged cohort of some 4,500 women followed up for an average of 5.9 years showed that more than half the cancers had not been notified within two and a half years of diagnosis, and that 14 had not been registered at all (Hunt & Coleman, 1987); this study was restricted to breast cancer, however, and involved a relatively small number of elderly women with an unrepresentative geographic distribution.

In this report, we provide an assessment of the cancer notification system using data from the Oxford-Family Planning Association (Oxford-FPA) study of contraception, in which 17,000 married women aged 25–39 years were recruited at 17 family planning clinics from 1968 to 1974, and flagged at the NHSCR in Southport. The women in the study have been followed up annually at the clinics, or by post, telephone or home visits, to collect data on the reasons for all hospital visits (inpatient or outpatient), on contraceptive practices and on several other related matters. Information has been provided voluntarily by each subject with a high standard of completeness and accuracy (Vessey *et al.*, 1974). Less than 10% of the women have been lost to follow up (including those who have emigrated) in the past 20 years. All self-reported cancer diagnoses have been confirmed by histology, and all diseases and deaths have been coded by one investigator (MV). The methods used in the Oxford-FPA study have been described in detail elsewhere (Vessey *et al.*, 1976). Direct ascertainment of malignant neoplasms in this large population, which had been flagged at the NHSCR since the inception of the current scheme in 1971, offered a unique opportunity to examine the completeness and timeliness of both cancer registration and notification.

### Population and methods

When the cohort was originally flagged at the NHSCR, 199 (1.2%) of 17,032 women were not traced. After provision of additional details in 1986, the entire cohort is now flagged.

A list was prepared of all malignant neoplasms reported by the women themselves to the Oxford-FPA study centre between May 1968 and 31 December 1985. *In situ* cancers were excluded because these tumours are known to be incompletely registered. All tumours have been coded to the 8th revision of the International Classification of Diseases since the start of the study. For each neoplasm, confirmation of the diagnosis was obtained from the appropriate medical practitioner as a copy of the pathology report.

This list was compared with the list of cancer notifications received from the NHSCR up to 30 June 1987. If no notification had been received, details were sent to the cancer registry corresponding to the woman's place of residence to discover if the cancer had been registered. Care was taken to ensure that the family name and address provided to the registry were those correct at the time of diagnosis of the cancer. Date of birth, cancer site, date of diagnosis and the

Correspondence: L. Villard-Mackintosh.
Received 17 February 1988; and in revised form, 7 June 1988.

name of the hospital and treating physician were also provided to ensure reference to the correct malignancy. Where the woman's address at diagnosis and the hospital of treatment were in different registry territories, both registries were contacted.

For tumours which had not been notified by the NHSCR but which had been duly registered at the regional cancer registry, we requested the date on which registrations for the relevant month or year were forwarded to OPCS. For those tumours which had not been registered, we attempted to discover why this was so.

It may be noted that the arrangement made with the NHSCR in Edinburgh for notification of cancers occurring in women resident in Scotland has been unsatisfactory and is under separate investigation. Further, only one woman in the study lived in Wales at the time of her cancer diagnosis. Accordingly, this report is restricted to the malignant neoplasms reported to us by women resident in England at the time of diagnosis, which were thus eligible for registration in England and eventual notification to use from the NHSCR in Southport.

Delays and incompleteness of cancer registration are examined by cancer site or type, by calendar period of diagnosis, and by cancer registry. For some of the notified tumours, the date of receipt of notification was recorded, and this can be compared with the date of diagnosis to examine the operating speed of the entire system.

### Results

Between May 1968 and the end of 1985, women in the study who were resident in England reported 325 malignancies. Breast cancer (150 cases) accounted for 46% of these tumours. The number of tumours rose steadily in successive quinquennia from 54 in 1970–74 to 96 in 1975–79 and 150 in 1980–84. By 30 June 1987, 219 (67%) of these tumours had been notified by the NHSCR. Of the remaining 106 neoplasms, 62 had been duly registered, all but three of which had been passed to OPCS, and 44 (13.5% of the total) had not been registered. Only two tumour notifications were received from the NHSCR for neoplasms which had not been ascertained from the women concerned. Table I gives the pattern of cancer registration and notification by site.

The proportion of tumours which had not been registered was a third or more for lung and pleura (ICD-8 162–3; 50% not registered), lymphosarcoma and other lymphoid neoplasms (200, 202; 38%), melanoma (172; 40%) and other neoplasms of skin (173; 36%), and bladder (188; 33%). Tumours at these sites accounted for 62 (19%) of those reported by women in the study. Among 150 breast cancers, 12 (8%) had not been registered, and for tumours of the digestive tract (ICD-8 150–7), ovary (183), eye and brain (191–2) and Hodgkin's disease (201), the proportion of unregistered tumours ranged from 5–14%. This group of tumours with less than 15% under-registration accounted for 203 (62%) of all tumours reported. For the mouth and pharynx (ICD-8 140–8), bone and connective tissue (170–1), cervix and body of uterus and other female genital organs (180–2, 184), thyroid gland (193) and leukaemia (myeloid only, 205), accounting for a further 55 (17%) of tumours reported, all tumours reported to us had been registered. There was no tendency for rare neoplasms to be systematically under-registered.

Table II shows the pattern of registration and notification by calendar period of diagnosis. The proportion of tumours that had not been registered increased from 7% in 1970–74 to 13% in both 1975–79 and 1980–84. Detailed examination of the data showed that this increase was not due to under-registration for any particular cancer site or at any particular tumour registry.

The proportion of all tumours ascertained from the women themselves that had also been notified to us by the NHSCR fell from 91% for 1970–74 to 76% for 1975–79, and to 59% in 1980–84. The time-lags inherent in the cancer registration scheme must be added to the time it takes NHSCR to process registrations and notify investigators, so that the proportion for 1985 (35% of 23 tumours) must be seen as provisional, but there is a clear downward trend in the proportion of all cancers ascertained directly which were first registered and later notified by the NHSCR across the three quinquennia 1970–84 (trend chi-square 21.85, 1-sided $P<0.001$).

If we consider only registered cancers, then the fall in the proportion notified is not so marked – 98%, 88% and 68% in successive quinquennia 1970–84 – but it is still large, and

**Table I** Malignant neoplasms in the Oxford-FPA study, 1968–85: Status of cancer registration at 30 June 1987, by site

| *ICD-8* | *Site* | *Total*[a] | *Notified (%)*[b] | *Not notified* OPCS[c] | *Not notified* Registry[d] | *Not notified* Not registered[e] No. (%) |
|---|---|---|---|---|---|---|
| 140–8 | Mouth and pharynx | 5 | 3 (60%) | 1 | 1 | |
| 150–7 | Digestive | 21 | 13 (62%) | 7 | | 1 (4.8%) |
| 162–3 | Respiratory | 6 | 1 (17%) | 1 | 1 | 3 (50.0%) |
| 170–1 | Connective | 5 | 5 (100%) | | | |
| 172 | Melanoma | 20 | 11 (55%) | 1 | | 8 (40.0%) |
| 173 | Other skin | 25 | 15 (60%) | 1 | | 9 (36.0%) |
| 174 | Breast | 150 | 104 (69%) | 33 | 1 | 12 (8.0%) |
| 180 | Cervix | 26 | 18 (69%) | 6 | | 2 (7.7%) |
| 181–2 | Uterus | 6 | 6 (100%) | | | |
| 183 | Ovary | 18 | 13 (72%) | 3 | | 2 (11.1%) |
| 184 | Other genital | 2 | 2 (100%) | | | |
| 188 | Bladder | 3 | 2 (67%) | | | 1 (33.3%) |
| 191–2 | Eye and brain | 7 | 5 (72%) | 1 | | 1 (14.3%) |
| 193 | Thyroid | 8 | 7 (88%) | 1 | | |
| 195–9 | Ill-defined, secondary | 4 | 3 (75%) | 1 | | |
| 200, 202 | Non-Hodgkin's lymphoma | 8 | 3 (38%) | 2 | | 3 (37.5%) |
| 201 | Hodgkin's disease | 7 | 6 (86%) | | | 1 (14.3%) |
| 205 | Myeloid leukaemia | 3 | 2 (67%) | 1 | | |
| 208 | Polycythaemia | 1 | 0 (0%) | | | 1 (100.0%) |
| | All sites | 325 | 219 (67%) | 59 | 3 | 44 (13.5%) |

[a]Total number of malignant neoplasms reported.
[b]Neoplasms registered and notified by the NHSCR to study investigators.
[c]Neoplasms registered and forwarded to OPCS.
[d]Neoplasms registered but not yet forwarded to OPCS.
[e]Number and percentage of all neoplasms at this site for which no registration was found.

**Table II** Malignant neoplasms in the Oxford-FPA study, 1968–85: Status of cancer registration at 30 June 1987, by period of diagnosis[a]

| *Calendar period of diagnosis* | *Total* | *Notified (%)* | *Not notified by June 1987* | | |
|---|---|---|---|---|---|
| | | | OPCS | *Registry* | *Not registered (%)*[b] |
| 1968–9 | 2 | 1 (50%) | 1 | | |
| 1970–4 | 54 | 49 (91%) | 1 | | 4 (7.4%) |
| 1975–9 | 96 | 73 (76%) | 10 | | 13 (13.5%) |
| 1980 | 25 | 14 (56%) | 8 | | 3 (12.0%) |
| 1981 | 21 | 12 (57%) | 5 | | 4 (19.0%) |
| 1982 | 34 | 22 (65%) | 9 | | 3 (8.8%) |
| 1983 | 35 | 21 (60%) | 11 | 1 | 2 (5.7%) |
| 1984 | 35 | 19 (54%) | 7 | 1 | 8 (22.9%) |
| 1985 | 23 | 8 (35%) | 7 | 1 | 7 (30.4%) |
| Total | 325 | 219 (67%) | 59 | 3 | 44 (13.5%) |

[a]See notes to **Table I**.
[b]Number and percentage of all tumours in each period not registered by 30 June 1987.

statistically significant ($P<0.001$). Overall, 62 tumours (22% of 281 registered) were not notified, and most of these had been diagnosed three or more years previously. For 130 (59%) of the 219 notifications received, the date of receipt had been noted, and although this had not been done systematically, the information was used to examine the distribution of time-lags between diagnosis of a cancer (anniversary date in the cancer registry) and its eventual notification. Table III shows that most notifications for which the date of receipt was recorded took between one and two years to reach the study; 84% of notifications arrived within four years and 93% within five years. The range was 4–87 months. The mean delay between diagnosis and notification fell from about four years in 1970–74 to 18 months in 1975–79, with an increase to two and a half years in the 1980–84. There was no major difference in delay between cancer sites.

The proportion of neoplasms not registered is shown for each registry in Table IV. Neoplasms ascertained directly were assigned to the registry in the territory of which the woman was living at the time of diagnosis. Overall, 13.5% of the 325 tumours were not registered. Excluding the (B) and (I) registries, in which less than 10 tumours were eligible for registration, under-registration ranged from 2% (D) to 42% (K). The women in the Oxford-FPA study are not a random sample of the female population of childbearing age, and more participating clinics drew their clientèle from the (C) region than any other. The sample of tumours is thus small for most individual registries, and three of the four registries at which 30 or more tumours were eligible for registration (C, D, J) had registered 90% or more of those tumours. However, under-registration was 42% (10/24) at (K) and 23% (10/43) at (H). Only three (7%) of the 44 unregistered tumours were treated in a cancer registry catchment area different from that of the woman's residence, but eight (18%) had been treated in private hospitals.

**Table III** Malignant neoplasms in the Oxford-FPA study, 1968–85: delay between diagnosis[a] and notification[b]

| *Delay (years)* | *No. (%)* |
|---|---|
| 0– | 13 (10%) |
| 1– | 36 (28%) |
| 2– | 39 (30%) |
| 3– | 21 (16%) |
| 4– | 12 (9%) |
| 5– | 8 (6%) |
| 6+ | 1 (1%) |
| Total | 130 (100%) |

[a]Anniversary date of the tumour in regional cancer registry.
[b]Date of notification by NHSCR to study investigators: this date was only recorded in Oxford for 130 (59%) of the 219 tumours notified by NHSCR.

**Table IV** Unregistered cancers by cancer registry of residence

| *Registry* | *Total*[a] | *Not registered (%)*[b] |
|---|---|---|
| A | 21 | 2 (9.5) |
| B | 5 | 0 (0.0) |
| C | 99 | 7 (7.1) |
| D | 41 | 1 (2.4) |
| E | 11 | 3 (27.3) |
| F | 20 | 3 (15.0) |
| G | 18 | 3 (16.7) |
| H | 43 | 10 (23.3) |
| I | 4 | 1 (25.0) |
| J | 39 | 4 (10.3) |
| K | 24 | 10 (41.7) |
| Total | 325 | 44 (13.5) |

[a]Total neoplasms reported in cohort by 31 December 1985.
[b]Not registered by 30 June 1987.

The 325 neoplasms were reported by 314 women, 11 of whom had true second primary malignant neoplasms. There were only 15 discrepancies between the ICD-8 site code assigned to the diagnosis reported by the woman (and confirmed by histology) and the site code supplied by OPCS: all but two were minor. Some notifications were either exact duplicates of a previous notification or differed in only minor detail (e.g., dates slightly different). Some of these appeared to arise from treatment of a recurrence. All the women in the study were married at the time of recruitment, but 31 (10%) of those with cancer have changed their name in the past 18 years, and 146 (47%) have changed their address, although only 13% moved to the catchment area of a different cancer registry.

### Discussion

Of 325 malignancies diagnosed in England among the women in this study between mid-1968 and the end of 1985, 44 had not been registered by June 1987, at least 1.5 years since diagnosis, suggesting overall completeness of registration of 86.5%. The estimate of completeness would be only slightly higher if a longer time were allowed between diagnosis and registration (at least 2.5 years, 87.8%; at least 3.5 years, 89.1%). Although a few unregistered tumours would probably have been registered eventually from the death certificate, they should be regarded as missed registrations in a system attempting to record incident neoplasms.

However, since the study population consisted only of married women of childbearing age, and was not evenly distributed among cancer registry territories, the figure of 86.5% is not a simple estimate of the completeness of female cancer registration in England. For example, only 11 (3%) of 325 cancers were diagnosed in the area covered by one of the larger registries, (E), which covers about 30% of the female population. Completeness of cancer registration and the size of population covered both vary considerably between the regional registries (OPCS, 1981). Several adjusted estimates were calculated to take account of the female population distribution between registries; these estimates differed by only 5% or so from the crude estimate, but they were sensitive to small changes in the criteria for inclusion of registries in the estimate (more than 10, 20 etc. tumours eligible for registration), and are not reported in detail.

The site distribution of tumours in this population is also somewhat atypical, with melanoma and breast cancer more common, and cancer of the cervix, uterus and bowel less common than in the general population, and since completeness of registration varies by site, this might also affect the estimate of completeness. However, with the proviso that the study population is not fully representative of all women in England by age, marital status, cancer risk or geographic distribution, the overall estimate of 86.5% for the completeness of female cancer registration in England seems reasonable. Cancers in this study population might be more readily diagnosed simply because the women were regularly attending family planning clinics, but these clinics do not provide information to cancer registries, and there is therefore no reason to suppose that cancers diagnosed in this population are more or less likely to be registered as a result of the woman being in the study than cancers in other women. It should be noted that the cancers recorded in this study were diagnosed over a 19-year period, but the estimate of completeness is heavily weighted by the period 1980–84, during which almost half the tumours were diagnosed (Table II).

We are not aware of any recent attempt at direct assessment of the completeness of cancer registration for England. Estimates for individual registries have been made: the most detailed of these suggest a level of ascertainment of about 94% for the N. Western registry (Nwene & Smith, 1982; Benn *et al.*, 1982). Other values have been given for Trent (close to 100%; Trout, 1982) and W. Midlands (98%; Waterhouse, 1982), but although levels of reporting in these registries are probably high, details of how these estimates were obtained are not given. On the basis of such estimates and the regional variation in the ratio of cancer deaths to cancer registrations, it has been suggested that in some regions completeness of ascertainment might be as low as 60–70 per cent (OPCS, 1981), although two registries acknowledged as defective in the past have since been reorganized and merged with the Thames registry. Donnan (1982) showed that at least part of the regional variation in cancer rates was likely to be due to deficient registration, but concluded that completeness of national ascertainment had increased between 1968 and 1976.

In Stockholm County, Sweden, completeness of registration in 1978 was estimated at 96% or more (Mattsson *et al.*, 1985) after direct examination of hospital record systems for over 6,000 cases first treated in 1978 and followed up for five years. This precise estimate refers to a single year for the main population centre (1.5 million) in a small country with highly developed health and registration systems. The estimate of completeness of registration provided here for England is based on a much smaller sample of cases, diagnosed over a long period of time, with an unrepresentative distribution by region, tumour site and age, and limited to females, but it is derived from direct ascertainment of malignant neoplasms from the subjects themselves, with histological confirmation.

Under-registration varied considerably by tumour site. Non-melanoma skin cancers are known to be under-registered, and nine (36%) of 25 were not registered in this population, but eight (40%) of 20 melanomas were not registered either, and this is surprising. The high proportions of unregistered tumours of lung (50%) and non-Hodgkin's lymphoma (38%) are based on only six and eight cases respectively. Only 12 (8%) of 150 breast cancers were not registered, compared with 14 (28%) of 50 breast cancers reported by Hunt & Coleman (1987). Cancer ascertainment in the larger study reported here is likely to have been more complete, however, and the figure of 8% for breast cancer is considered more reliable.

The only other distinguishing feature of the unregistered tumours was that eight (18%) were treated in private hospitals. There is already some concern that cancers in patients treated privately are not being registered (Balarajan & Scott, 1983) and a similar proportion (21%) of privately treated patients was observed among unregistered breast cancers reported by Hunt & Coleman (1987), although this was based on small numbers.

The accuracy of cancer registration would appear high. Only two site-codes assigned to tumours within this study and at cancer registration differed at the third digit of ICD-8, and there was one major error in date of diagnosis; minor differences in dates of birth or of diagnosis were common, however. Some cancers were notified twice, although the later notification was clearly for a recurrence of the original tumour; only 11 true second primary neoplasms were recorded.

Before an investigator can learn of a cancer from the national registration scheme, it must be diagnosed, recorded in the regional cancer registry, forwarded to the OPCS national registry, passed to the NHSCR, flagged there and finally notified to the investigator. There is clearly potential for error and delay in this system, and it is worth considering the fate of the 281 cancer registrations detected during this exercise. There was a marked decline in the proportion of registrations notified to us over a 15-year period, from 98% in 1970–74 to 68% in 1980–84, though only about 50% of notifications arrived within 2.5 years of diagnosis (Table III), and the final proportion for 1980–84 may eventually be higher than 68%. Overall, 219 (78%) of cancer registrations were notified to us, whereas over 98% of all cancer registrations sent to NHSCR were apparently flagged for the period 1971–78, with very little variation between consecutive years or between different registries (OPCS, unpublished tables). These observations are difficult to reconcile, unless either (a) a proportion of tumour registrations received by OPCS from the regional registries is not forwarded to the NHSCR, or (b) the final step of notifying the investigator is omitted in a proportion of cases.

The timeliness of notification has improved since the system of flagging began in 1971. Many tumours diagnosed in the early 1970s were only notified four years later, and the range of delays was wide (4–87 months). More recently, mean lag-times have been shorter (1.5 years in 1975–79 and 2.5 years in 1980–85) and the range narrower (12–52 months). It should be noted that a large number of cohorts is currently flagged at the NHSCR, involving many thousands of subjects (see OPCS, 1981). As Donnan (1982) has pointed out, the staff engaged in tracing cancers in flagged cohorts and notifying them to the investigators are also employed for updating the National Cancer Register, and the combined workload is considerable.

It is perhaps worth emphasizing our opinion that the notification service for deaths and cancer registrations provided since 1971 by the National Health Service Central Register has been and remains of immense value in many epidemiological studies involving long-term follow-up of large numbers of persons, providing for most of them crucial information which could often not have been obtained, as was possible in this study, in any other way. The particular design adopted for this study made it possible for the efficiency of the notification scheme to be assessed, and the twin purpose in presenting these results is first, to provide other investigators who use the scheme with information about it which is not usually available, and second, to assist the Office of Population Censuses and Surveys, which operates the scheme, to identify and correct its weaknesses.

Since investigators can only be notified of a cancer by the NHSCR if it has first been registered, the variable degree of completeness of cancer registration between the different regional registries should also be considered in the interpretation of studies using the NHSCR for ascertainment of cancers. Our estimate of 86.5% for the completeness of cancer registration in England strictly applies only to women of childbearing age; it should be interpreted in the light of unrepresentative geographic distribution of subjects and the long time period covered, although most of the cancers involved were diagnosed since 1980. It might be valuable if direct assessment of the completeness of cancer registration could also be derived from other large cohort studies in which cancers have been ascertained independently of the cancer registration scheme.

We thank Mrs C. Brice, Mrs P. Brown, Dr D. Yeates, and our research assistants in the participating clinics for their continued loyal support. We gratefully acknowledge the cooperation we have received from both the National Health Service Central Register in Southport and from all the cancer registries included in this report. We also thank the Medical Research Council for financial support.

### References

BALARAJAN, R. & SCOTT, A. (1983). National cancer registration: An appraisal. *Comm. Med.*, **5**, 31.

BENN, R.T., LECK, I. & NWENE, U.P. (1982). Estimation of completeness of cancer registration. *Int. J. Epidemiol.*, **11**, 362.

DONNAN, S. (1982). Cancer registration – advance or retreat? In *Recent Advances in Community Medicine*, 2. Smith, A. (ed), p. 157. Churchill Livingstone: Edinburgh.

HUNT, K. & COLEMAN, M.P. (1987). The completeness of cancer registration in follow-up studies – A cautionary note. *Br. J. Cancer*, **56**, 357.

MATTSSON, B., RUTQVIST, L.E. & WALLGREN, A. (1985). Under-notification of diagnosed cancer cases to the Stockholm Cancer Registry. *Int. J. Epidemiol.*, **14**, 64.

NWENE, U. & SMITH, A. (1982). Assessing completeness of cancer registration in the North-western region of England by a method of independent comparison. *Br. J. Cancer*, **46**, 635.

OFFICE OF POPULATION CENSUSES AND SURVEYS (1981). *Report of the Advisory Committee on Cancer Registration: Cancer Registration in the 1980s*. Series MB1, no. 6. HMSO: London.

SWERDLOW, A.J. (1986). Cancer registration in England and Wales: Some aspects relevant to interpretation of the data. *J. R. Statist. Soc. A.*, **149**, (part 2), 146.

TROUT, K. (1982). UK, England, Trent Region. In *Cancer Incidence in Five Continents* (IV), Waterhouse, J. *et al.* (eds), p. 566. IARC: Lyon.

VESSEY, M., DOLL, R., PETO, R., JOHNSON, B. & WIGGINS, P. (1976). A long-term follow-up study of women using different methods of contraception – An interim report. *J. Biosoc. Sci.*, **8**, 373.

VESSEY, M.P., JOHNSON, B. & DONNELLY, J. (1974). Reliability of reporting by women taking part in a prospective contraceptive study. *Br. J. Prev. Soc. Med.*, **28**, 104.

WATERHOUSE, J.A.H. (1982). UK, England, Birmingham and West Midlands Region. In *Cancer Incidence in Five Continents* (IV), Waterhouse, J. *et al.* (eds), p. 550. IARC: Lyon.


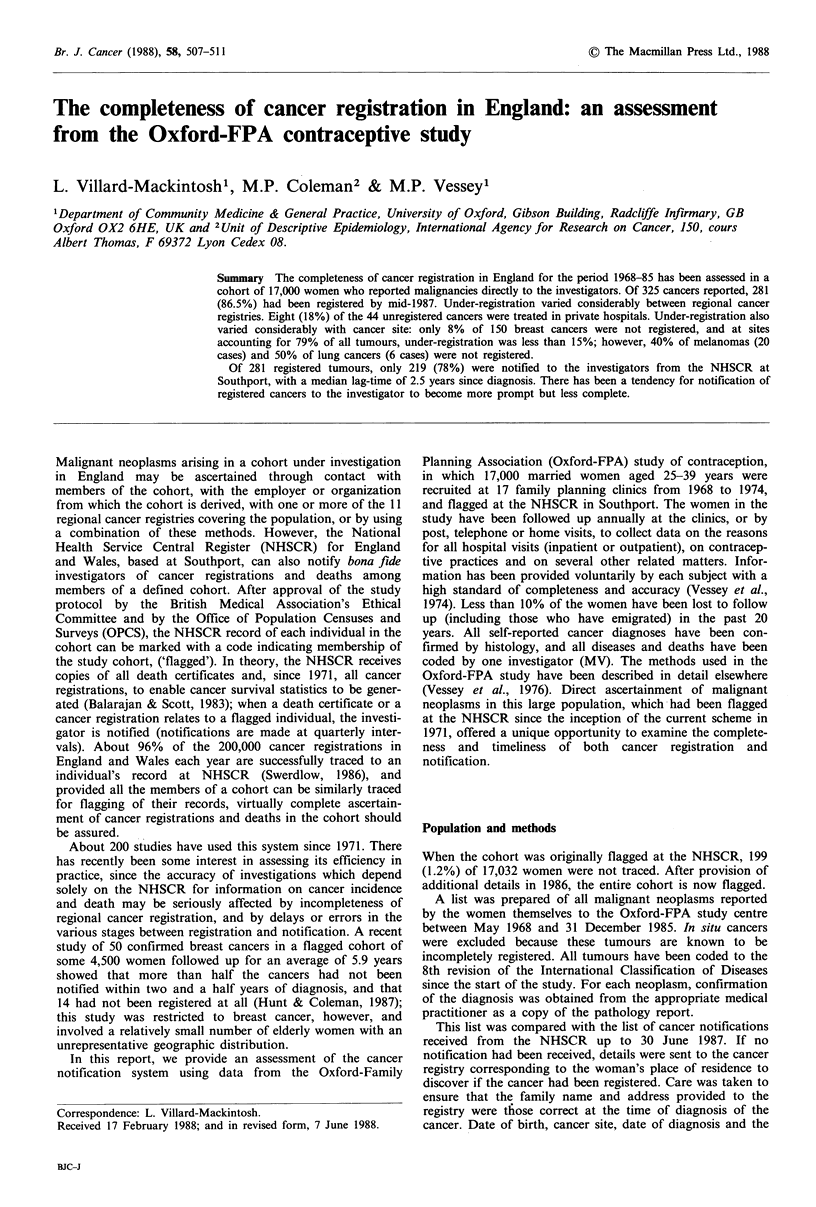

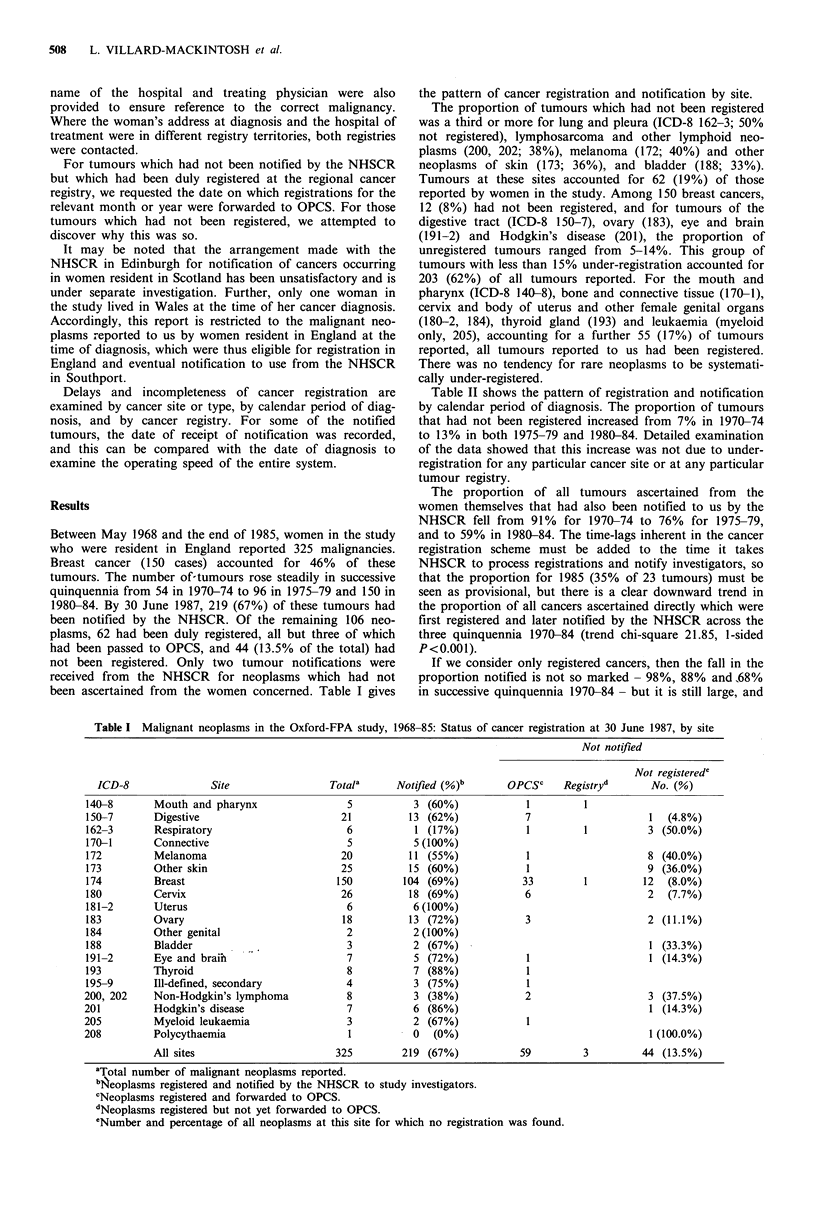

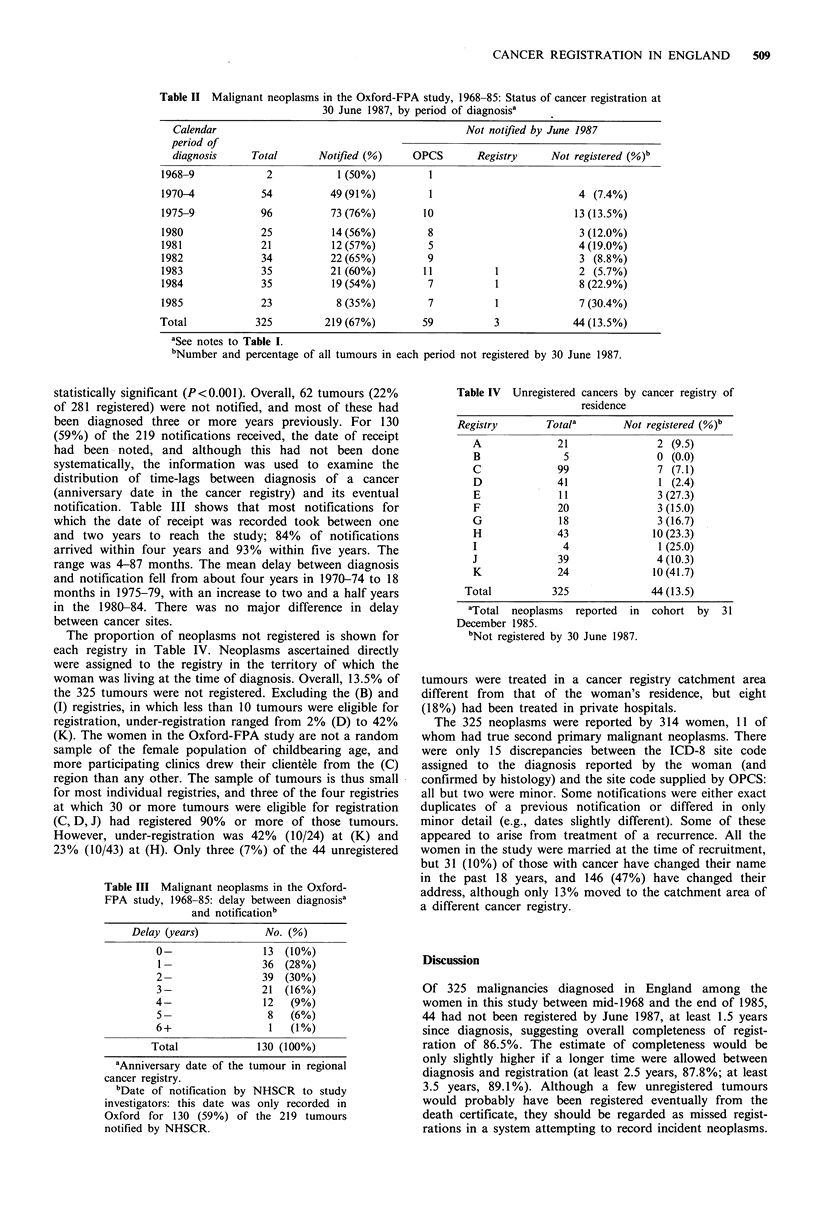

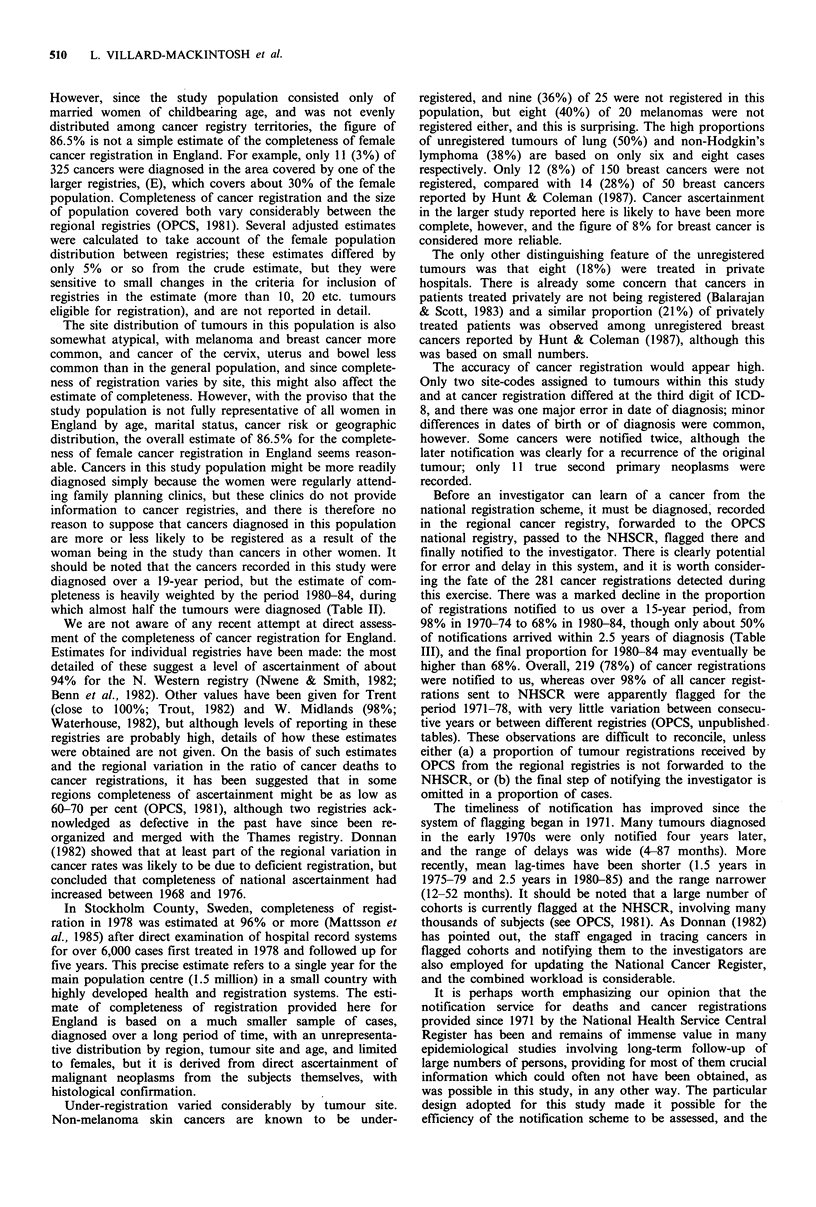

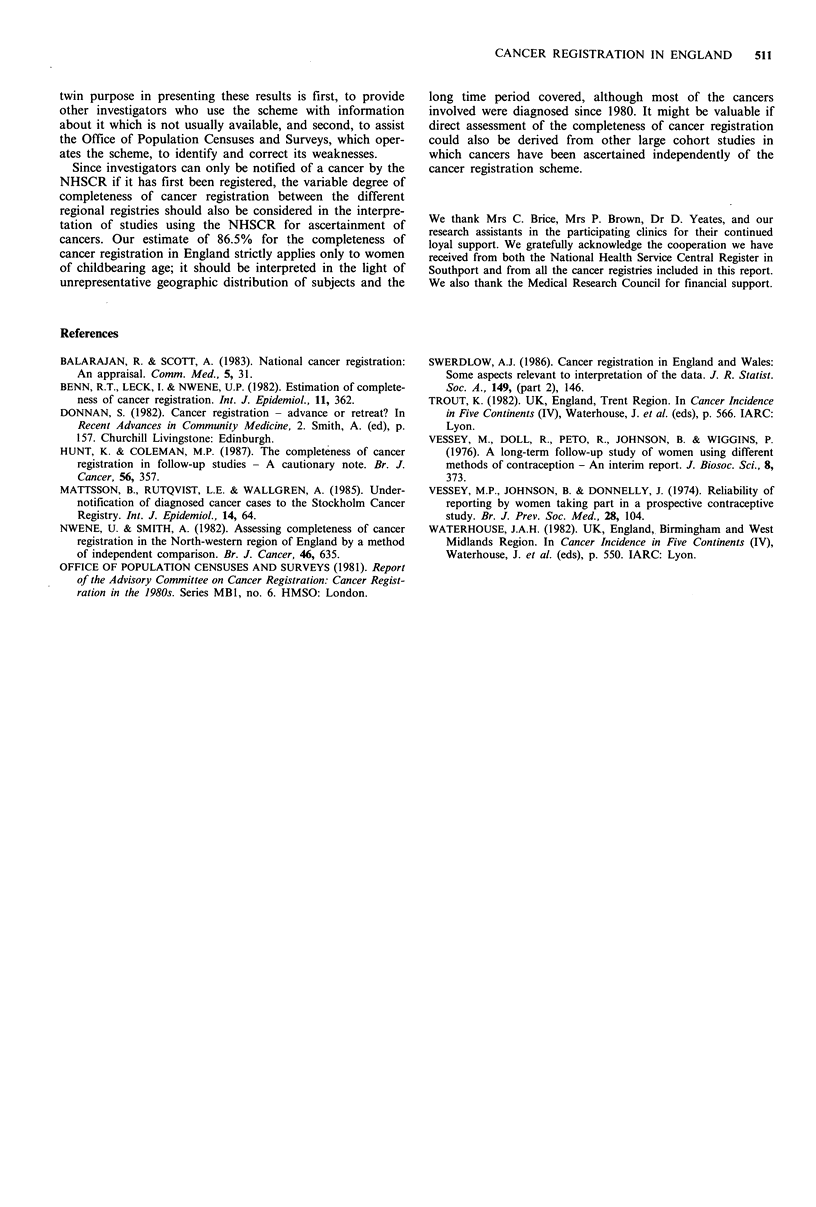


## References

[OCR_00774] Balarajan R., Scott A. (1983). National cancer registration: an appraisal.. Community Med.

[OCR_00778] Benn R. T., Leck I., Nwene U. P. (1982). Estimation of completeness of cancer registration.. Int J Epidemiol.

[OCR_00787] Hunt K., Coleman M. P. (1987). The completeness of cancer registration in follow-up studies--a cautionary note.. Br J Cancer.

[OCR_00792] Mattsson B., Rutqvist L. E., Wallgren A. (1985). Undernotification of diagnosed cancer cases to the Stockholm Cancer Registry.. Int J Epidemiol.

[OCR_00797] Nwene U., Smith A. (1982). Assessing completeness of cancer registration in the north-western region of England by a method of independent comparison.. Br J Cancer.

[OCR_00823] Vessey M. P., Johnson B., Donnelly J. (1974). Reliability of reporting by women taking part in a prospective contraceptive study.. Br J Prev Soc Med.

[OCR_00817] Vessey M., Doll R., Peto R., Johnson B., Wiggins P. (1976). A long-term follow-up study of women using different methods of contraception--an interim report.. J Biosoc Sci.

